# Hydrophilic Interaction Liquid Chromatography-Electrospray Ionization Mass Spectrometry for Therapeutic Drug Monitoring of Metformin and Rosuvastatin in Human Plasma

**DOI:** 10.3390/molecules23071548

**Published:** 2018-06-27

**Authors:** Nikolaos Antonopoulos, Giorgos Machairas, George Migias, Ariadni Vonaparti, Vasiliki Brakoulia, Constantinos Pistos, Dimitra Gennimata, Irene Panderi

**Affiliations:** 1 Laboratory of Pharmaceutical Analysis, School of Pharmacy, Division of Pharmaceutical Chemistry, National and Kapodistrian University of Athens, Panepistimiopolis, Zografou, 15771 Athens, Greece; nikos_antono@windowslive.com (N.A.); giorgosmachairas@yahoo.com (G.M.); vasilikibrakoulia@gmail.com (V.B.); 2 General Hospital of Athens Alexandra, 80 Vas. Sofias Avenue, 11528 Athens, Greece; g.migias@gmail.com; 3 Qatar Doping Analysis Laboratory, Sports City Road, Aspire Zone, P.O. Box 27775, Doha, Qatar; avonaparti@adlqatar.qa; 4 Department of Chemistry, West Chester University, West Chester, PA 19383, USA; cpistos@wcupa.edu; 5 General Hospital Korgialenio-Benakio National Red Cross, Erithrou Stavrou 1, 11526 Athens, Greece; dimigenn@gmail.com

**Keywords:** rosuvastatin, metformin, HILIC, LC-MS, therapeutic drug monitoring

## Abstract

In this work a hydrophilic interaction liquid chromatography/positive ion electrospray mass spectrometric assay (HILIC/ESI-MS) has been developed and fully validated for the quantitation of metformin and rosuvastatin in human plasma. Sample preparation involved the use of 100 μL of human plasma, following protein precipitation and filtration. Metformin, rosuvastatin and 4-[2-(propylamino) ethyl] indoline 2 one hydrochloride (internal standard) were separated by using an X-Bridge-HILIC BEH analytical column (150.0 × 2.1 mm i.d., particle size 3.5 μm) with isocratic elution. A mobile phase consisting of 12% (*v*/*v*) 15 mM ammonium formate water solution in acetonitrile was used for the separation and pumped at a flow rate of 0.25 mL min^−1^. The linear range of the assay was 100 to 5000 ng mL^−1^ and 2 to 100 ng mL^−1^ for metformin and rosuvastatin, respectively. The current HILIC-ESI/MS method allows for the accurate and precise quantitation of metformin and rosuvastatin in human plasma with a simple sample preparation and a short a chromatographic run time (less than 15 min). Plasma samples from eight patients were further analysed proving the capability of the proposed method to support a wide range of clinical studies.

## 1. Introduction

Diabetes mellitus is a metabolic disorder caused by failures in the action and/or secretion of insulin. During the last decades, incidents of diabetes have increased from 108 million to 422 million corresponding mainly in type 2 diabetes mellitus [1]. Diabetes mellitus is a risk factor for a wide range of vascular diseases like ischaemic stroke and coronary heart disease [2]. Metformin belongs to a class of biguanides and it is usually administered for non-insulin-dependent type II diabetes mellitus as the first line of treatment when lifestyle modification alone is not enough [3]. Several studies in the literature report the beneficial protective effects of metformin administration in cardiac function, resulting in the deterioration of mortality caused from cardiovascular deceases as a result of type 2 diabetes mellitus [4]. For the treatment of patients with type 2 diabetes mellitus it is often required drug therapy with beneficial effects on the lowering of blood glucose and on dyslipidemia. Statins are administered for the prevention of cardiovascular diseases and are also effective to prevent vascular events in diabetic patients [5]. Rosuvastatin is a 3-hydroxy-3-methylglutaryl-coenzyme A (HMG-CoA) reductase inhibitor that is administered for hypercholesterolemia in patients with a high risk of developing atherosclerosis and in patients with established cardiovascular disease [6]. 

Metformin and rosuvastatin are frequently prescribed in combination due to the high comorbidity of these two diseases [7]. The development of novel analytical methods for therapeutic drug monitoring is crucial to support the model of personalized medicine and the introduction of new drug combinations in therapy. Thus, the purpose of this study was on the development of a new method to detect and quantify those two commonly prescribed drugs in human plasma.

Several methods have been reported in literature for the quantitation of metformin alone [8,9,10,11,12] or in combination with other drugs [13,14,15,16,17,18,19,20,21] in biofluids. A number of other methods have also been reported in literature [22] for the quantitation of rosuvastatin alone [23,24,25,26,27,28,29,30] or in combination with other drugs [31,32] in human plasma. The pharmacokinetic parameters of metformin and rosuvastatin in healthy volunteers have been studied by the use of two different reversed-phase liquid chromatography tandem mass spectrometric methods procedures for each analyte and complicated sample preparation procedures involving liquid-liquid extraction [7]. A study on the pharmacokinetics of rosuvastatin after concomitant administration of metformin and/or furosemide was conducted by using two different RP-LC tandem mass spectrometric methods. The first method was used in combination with a solid phase extraction procedure for the determination of metformin alone and the second one in combination with a liquid-liquid extraction for the determination of rosuvastatin and furosemide combination [33]. Recently, a reversed-phase tandem mass spectrometric method was developed and fully validated for the simultaneous determination of metformin and rosuvastatin in human plasma in combination with a simple sample preparation procedure [34]. Most recently a HILIC method coupled to diode array detection has been published by our group for the quantitation of rosuvastatin and metformin impurities in fixed-dose combination tablets containing rosuvastatin and metformin [35]. To the extent of our knowledge, no bioanalytical assay has been previously developed for the simultaneous quantitation of metformin and rosuvastatin in human plasma by hydrophilic interaction liquid chromatography (HILIC) and by using a single quadrupole mass spectrometer. HILIC offers improved sensitivity when combined with mass spectrometric detection due to the high percentage of organic solvents used in the mobile phase in order to elute polar compounds. The proposed method is suitable for therapeutic drug monitoring of metformin and rosuvastatin and it has been successfully applied to the analysis of clinical samples obtained from patients that have been treated with the analysed drugs.

## 2. Results and Discussion

### 2.1. Method Development

#### 2.1.1. MS Detection Optimization

**Figure 1:**
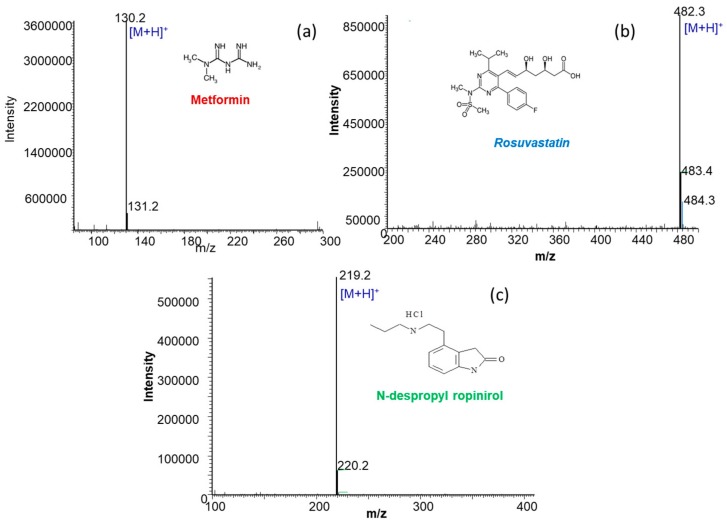
ESI mass spectra in positive ion mode of a 10 μg mL^−1^ solution prepared in mobile phase of (**a**) metformin (**b**) rosuvastatin and (**c**) N-despropyl ropinirole.

The parameters for electrospray ionization (ESI) have been optimized so as to allow maximum abundance of the molecular ions of metformin, rosuvastatin and the ISTD. The mass spectrometer was operated in positive ESI ion mode and selected ion monitoring (SIM) was used to analyze the protonated molecules [M + H]^+^ at *m/z* 130, 482 and 219 for rosuvastatin, metformin and N-despropyl ropinirole (ISTD), respectively. The maximum abundance of the selected ions was achieved when capillary voltage, mass span and dwell time were set at 4.8 kV, 0.1 and 0.3 s, respectively. Mass spectra of metformin, rosuvastatin and N-despropyl ropinirole (ISTD) are presented in Figure 1.

#### 2.1.2. Chromatography

The chromatographic separation of compounds with a broad band of polarity can be easily achieved by using HILIC chromatography [36]. The separation mechanism in HILIC combines partition, reversed-phase interactions, normal phase/adsorption, electrostatic interactions and hydrogen bonding and thus it is ideal to separate compounds with diverse physicochemical properties [37]. Since metformin and rosuvastatin have sufficiently different physicochemical properties a zwitterionic ZIC^®^-pHILIC analytical column has been used in preliminary experiments to separate these compounds. In most of the mobile phases that have been tested rosuvastatin was eluted very close to the solvent front even when the aqueous content of the mobile phase was reduced below 6%. Thus, a hydrophilic XBridge^®^-HILIC BEH analytical column was chosen in this work as the most suitable for the chromatography. The packing material of this column consists of two kind of monomers, bis-triethoxysilylethane and tetraethoxysilane, which are joined with ethylene bridges. Some accessible silanols that remain on the surface of these BEH particles are responsible for electrostatic interactions. Metformin is 100% positively-charged over a pH range from 1 to 12, while rosuvastatin is 100% negatively-charged at pH values above 6. The internal standard, N-despropyl ropinirol is 100% positively-charged over a pH range from 1 to 7.5. Previous findings, of our research group [35], pointed out that the mechanism of separation for rosuvastatin and metformin in HILIC comprises both secondary electrostatic interactions and hydrophilic partition. 

**Figure 2:**
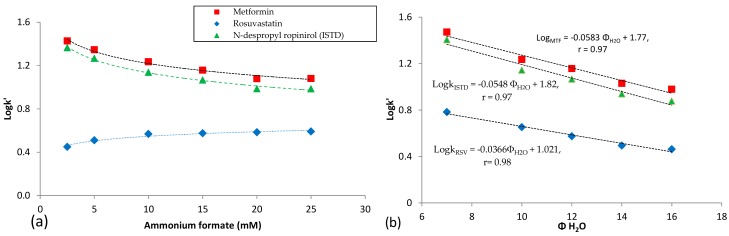
Plots of logk values for rosuvastatin, metformin and N-despropyl ropinirole versus (**a**) the concentration of ammonium formate, and (**b**) the percentage of the water of the mobile phase on an XBridge^®^-HILIC BEH analytical column. Data have been obtained from the analysis of human plasma samples spiked with 2500 ng mL^−1^ metformin, 50 ng mL^−1^ rosuvastatin and 190 ng mL^−1^ N-despropyl ropinioro (ISTD).

Several combinations of the mobile phase constituents, acetonitrile asorganic modifier and aqueous ammonium formate buffer, have been tested to optimize the chromatographic parameters in order to achieve adequate separation of the analytes from matrix interferences and reach the best sensitivity in ESI-MS detection. With a constant water content of the mobile phase eluent at 12%, the concentration of the aqueous ammonium formate buffer was varied from 2.5 to 25 mM. Figure 2a shows that the retention of both metformin and N-despropyl ropinirol is decreased by increasing the concentration of ammonium formate up to 25 mM, while the retention of rosuvastatin is increased. Based on the above findings it can be concluded for metformin and ISTD, that by increasing the buffering salt concentration the electrostatic attraction between the positively-charged compounds and the negatively-charged silanol groups of the XBridge^®^-HILIC BEH particle is reduced. On the contrary, for rosuvastatin the slight increase in retention by increasing the buffering salt concentration reduces the electrostatic repulsion of the negatively charged rosuvastatin molecule and the negatively charged silanol groups of the XBridge^®^-HILIC BEH particles. These results have been obtained from the analysis of human plasma samples spiked with 2500 ng mL^−1^ metformin, 50 ng mL^−1^ rosuvastatin and 190 ng mL^−1^ of N-despropyl ropinirol (ISTD). It was also observed that by reducing the concentration of ammonium formate below 10 mM the peak shape of metformin and N-despropyl ropinirole was distorted and split. Thus, a 15 mM aqueous ammonium formate concentration was chosen as the optimum.

**Figure 3:**
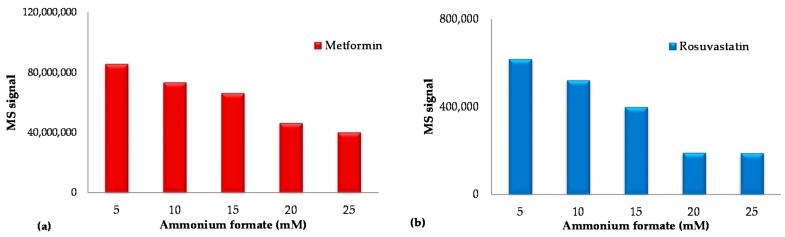
Impact of the concentration of ammonium formate in the mobile phase on the MS signal of (**a**) metformin and (**b**) rosuvastatin. Data have been obtained from the analysis of human plasma samples spiked with 2500 ng mL^−1^ metformin, 50 ng mL^−1^ rosuvastatin and 190 ng mL^−1^ N-despropyl ropinioro (ISTD).

The effect of the percentage of water, φ_water_, was also evaluated in experiments where φ_water_ was varied from 7 to 16% while the concentration of ammonium formate was kept constant at the optimum value of 1.8 mM (pH = 6.5) in whole mobile phase. As it can be observed in Figure 2b, the retention of the analytes decreases linearly with increasing the percentage of water, implying a partition mechanism in HILIC separation. It was also observed an improvement in ESI/MS sensitivity for metformin (Figure 3a) and rosuvastatin (Figure 3b) by decreasing the concentration of the buffering salt in the mobile phase, while the percentage of water, φ_water_, was kept constant at 12% (*v*/*v*). 

Based on the above studies, the optimum mobile phase composition consists of 12% 15 mM ammonium formate water solution pH 6.5 in acetonitrile. The proposed HILIC-ESI/MS method allows the isocratic separation of the analytes with 15 min. After column equilibration time of 1 h a great number of samples can be analysed within one analytical daily batch. The internal standard, N-despropyl ropinirol, exhibits adequate ion intensity and good chromatographic peak shape under the selected chromatographic and MS conditions. This compound is an impurity of ropinirol, thus, it cannot be found in human plasma of patients. 

**Figure 4:**
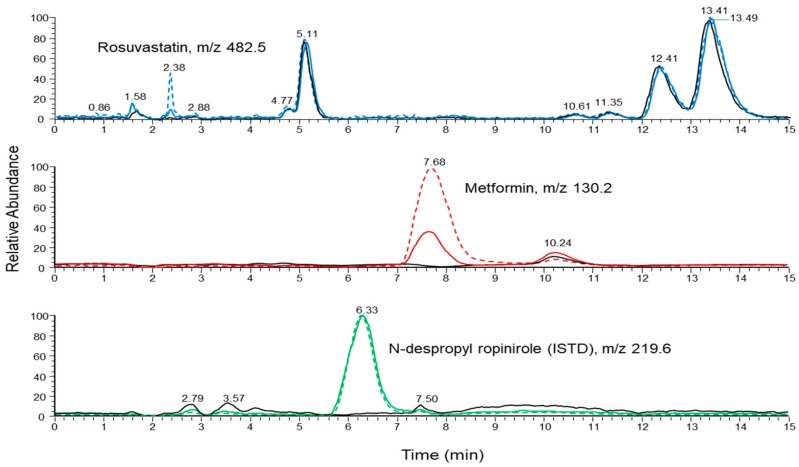
Ion chromatogram (SIM mode) of a blank human plasma sample (black line) overlaid with ion chromatograms (SIM mode) of calibration spiked plasma samples at 2 ng mL^−1^ (solid line) and 20 ng mL^−1^ (dashed line) of rosuvastatin (blue line), 100 ng mL^−1^ (solid line) and 1000 ng mL^−1^ (dashed line) of metformin (red line) and 190 ng mL^−1^ of ISTD (green line).

The selectivity of the proposed method is illustrated in Figure 4, where a representative ion chromatogram of a blank plasma sample is overlaid with ion chromatograms of calibration plasma samples spiked with 100 and 1000 ng mL^−1^ of metformin and 2 and 20 ng mL^−1^ of rosuvastatin, respectively and 190 ng mL^−1^ of N-despropyl ropinirol (ISTD). Rosuvastatin, metformin and N-despropyl ropinirol were eluted at 2.4, 7.7 and 6.3 min, respectively.

#### 2.1.3. Optimization of the Preparation of the Biological Sample

Protein precipitation is a simple sample preparation procedure that allows for the analysis of a large number of samples in short time. In this work protein precipitation gave adequate recovery for metformin, rosuvastatin and the ISTD, while further filtration was applied to minimize the matrix interference, and to prolong the column life time and prevent system blockages [38]. In HILIC the nature of the dissolution solvent is more crucial than in reversed-phase HPLC on the chromatographic peak shape. It was observed during method development that the peak shapes of metformin and rosuvastatin were split and distorted in different dissolution solvent mixtures and by changing the percentage of the biological sample in the injection sample. 

**Figure 5:**
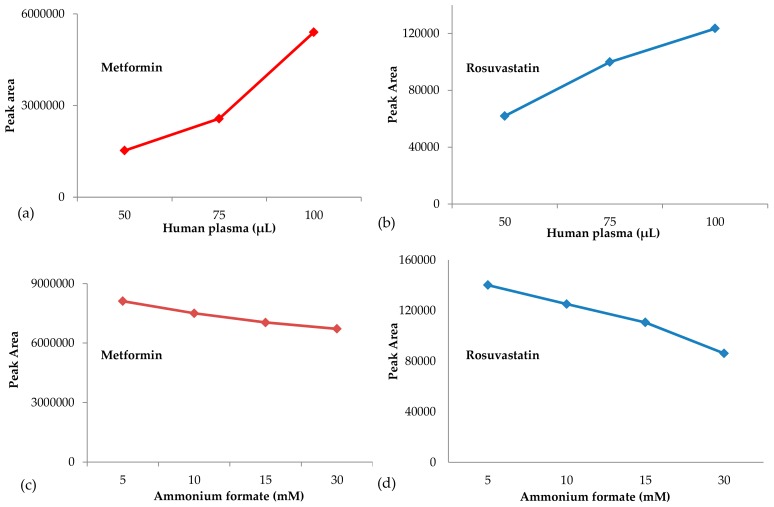
Effect of the proportion of the biological sample (**a** and **b**) and the concentration of a 30% (*v*/*v*) ammonium formate water solution in acetonitrile (**c** and **d**) on the peak area signal of metformin and (**b**,**d**) rosuvastatin.

Various proportions of human plasma have been tested to determine the optimum amount of biological material for the analyses. Figure 5 shows that by increasing the amount of human plasma up to 100 μL, peak area signal of metformin (Figure 5a) and rosuvastatin (Figure 5b) was increased. A further increase of human plasma above 100 μL caused serious distortion of the peak shapes of the analytes. Thus, analysis was performed by using 100 μL of biological sample. To further improve the chromatographic peak shapes, buffered eluents have been used. Therefore, we tested different concentrations of 30% (*v*/*v*) ammonium formate water solution in the dissolution solvent. Results presented in Figure 5c,d, for metformin and rosuvastatin, respectively, indicate that peak area signal of rosuvastatin decreases by increasing the concentration of ammonium formate. We concluded that the best peak shapes were obtained by using 100 μL of biological sample and processed with a dissolution solvent mixture consisting of 30% (*v*/*v*) of an ammonium formate water solution at 5 mM (pH 6.5) in acetonitrile. A membrane syringe filter was also used for the filtration of the samples prior to HILIC-ESI/MS analysis. Filtration was essential to remove any additional matrix interferences and increase the sensitivity in HILIC-ESI/MS system. To evaluate whether filtering of the processed biological samples would cause loss of the analytes, eight different types of syringe filters were selected for testing, five 0.45 μm pore size and three 0.22 μm pore size, all 13 mm diameter. 

**Figure 6:**
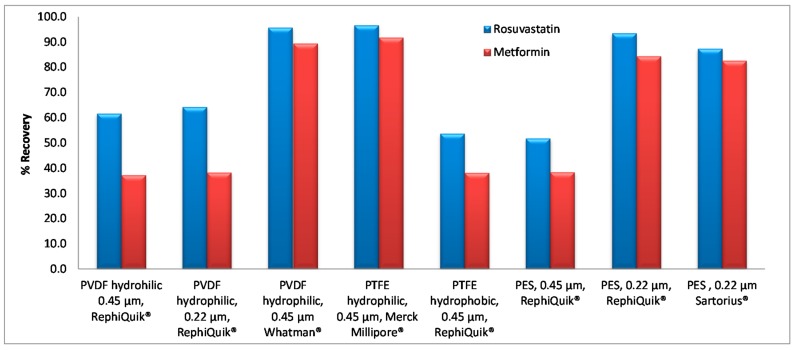
Effect of the type of the filters used for sample preparation of the biological samples on the % recovery of the analytes.

To evaluate the % recovery, the peak area of spiked plasma samples was compared with the analytical response of blank plasma samples spiked with the equivalent concentration of the analytes after the sample preparation and filtration procedure. Data presented in Figure 6, shows the % recovery of the analytes under the various types of the filters that have been tested. 

Optimum % recovery for metformin and rosuvastatin was achieved when a 100 μL aliquot of each human plasma sample was processed by the addition of 50 μL of ISTD solution (3.8 μg mL^−1^), 30 μL of 5 mM ammonium formate solution in water and 820 μL of acetonitrile. After vortex mixing and centrifugation, the supernatant was filtered through a 13 mm PTFE membrane syringe filter (hydrophilic) with a pore size of 0.45 μm.

### 2.2. Statistical Analysis of Data

#### 2.2.1. Selectivity and Specificity

The selectivity test met the pre-defined criteria as co-eluting peaks where less than 5% of the area of metformin and rosuvastatin at the LOQ level, and less than 5% of the area of N-despropyl ropinirole in the ion chromatograms (SIM mode) obtained from the analysis of six batches of human plasma. The carry-over test has been performed by analysing blank human plasma samples after the injection of the highest concentration calibration and showed no interfering peaks with peak areas greater than 5% of the peak areas at the LOQ level of metformin and rosuvastatin.

#### 2.2.2. Linearity, Precision and Accuracy

A weighting factor of 1/y^2^ was used for the linear regression analysis and data presented in Table 1, indicate that linear relationships have been achieved between the measured signal ratios of the analytes and the corresponding concentrations. In agreement with international guidelines [39], the back-calculated concentrations in the calibration curves were less than 11.5% and 14.5% of the nominal concentration of metformin and rosuvastatin.

The LOD and LLOQ were estimated from the signal-to-noise ratio (S/N) and by analysing human plasma samples spiked with metformin and rosuvastatin at low concentrations. The LOD was estimated at a S/N ratio of 3:1 and the LLOQ was estimated at a S/N ratio of at least 10:1, until a % CV of less than 20% was obtained. Τhe LODs were found to be at 11 and 0.3 ng mL^−1^, and the LLOQs at 35 and 1 ng mL^−1^, for metformin and rosuvastatin, respectively. 

Precision and accuracy data are presented in Table 2 and indicate that intra-assay coefficients of variations, % CV, were between 5.3% and 9.1% for metformin and between 6.2% and 8.5% for rosuvastatin. The inter-assay % CVs were lower than 3.0% and 7.1% for metformin and rosuvastatin, respectively. The overall accuracy, which was assessed by the percentage relative error, was ranged from −3.3 to 0.4% for metformin and from −1.1 to 7.0% for rosuvastatin. 

#### 2.2.3. Recovery and Matrix Effect

Recovery data are presented in Table 3 and indicate average recovery of more than 92.6% and 95.8% for metformin and rosuvastatin, respectively. The average recovery for N-despropyl ropinirol (ISTD) was found to be 91.7 ± 3.8%. 

The method described by Matuszewski et al. [40] was used to evaluate the matrix effect for metfromin, rosuvastatin and the ISTD. The matrix effect was expressed by the percentage matrix factor which is defined as the percentage of the ratio of the analyte peak response in the presence of matrix ions to the analyte peak response in the absence of matrix ions. As shown by the results presented in Table 4, the % matrix factor was greater than 59.9% and 81.3% for metformin and rosuvastatin, respectively indicating low ion suppression. The % matrix factor for N-despropyl ropinirol (ISTD) was found to be 61.8 ± 1.8%. 

#### 2.2.4. Stability

The stability of the analytes in spiked human plasma samples was evaluated under various storage conditions presented in detail in Table 4. To evaluate stability the results of the stored samples were compared to the results of the freshly prepared samples spiked with the analytes in human plasma at equivalent concentrations. 

Stability results are presented in Table 4 and indicate that the concentrations of metformin and rosuvastatin deviate by no more than −2.6% relative to the reference for any of the analytes. Thus, human plasma samples containing metformin and rosuvastatin may be kept without any substantial degradation under the various tested storage conditions.

### 2.3. Application to the Analysis of Real Samples

Human plasma samples collected from patients that were treated with the analytes have been analysed by the current method so as to demonstrate the applicability of the method to support several clinical studies related to metformin and rosuvastatin therapy. Human plasma samples collection was approved by the local Ethics Committee of clinical settings, although not demanded by national legislation, as this study is not regarded as a clinical trial.Blood samples were collected in tubes containing sodium heparin as anticoagulant and immediately after drawing, the samples were shaken gently and centrifuged at 4000 rpm for 10 min at 4 °C. Human plasma samples were stored at −20 °C and analysed within two weeks after storage by the proposed HILIC-ESI/MS method. In particular, plasma samples obtained from eight human patients (five female and three male) with ages ranging from 57 to 88 years old have been analysed by the proposed method. During the period of sample collection, all of the patients had receiving other medication described in detail in Table 5. 

As shown by the clinical data presented in Table 5, rosuvastatin was quantitated in human plasma samples obtained from two male patients who had receiving 20 mg of rosuvastatin once daily in the morning (Crestor^®^ 20, AstraZeneca A.E., Cambridge, UK) for a long-term treatment and blood samples were collected at 11 and 16 h after dosing. The concentrations of rosuvastatin ranged from 9.21 ± 0.39 to 16.2 ± 1.4 ng mL^−1^. 

Metformin was quantitated in human plasma samples obtained from five patients (four female and one male) who had receiving either 1000 mg of metformin (Glucophage^®^ 1000, Merck A.E., Kenilworth, NJ, USA) once daily in the morning or 850 mg of metformin twice daily (Glucophage^®^ 850, Merck A.E.) and for a long-term treatment. The concentrations of metformin ranged from 226 ± 12 to 2635 ± 67 ng mL^−1^. 

Rosuvastatin and metformin were quantitated in one female patient who had receiving 1000 mg of metformin once daily (Glucophage^®^ 1000, Merck A.E.) and 10 mg of rosuvastatin once daily (Crestor^®^ 10, AstraZeneca A.E.). Plasma sample obtained from this patient exhibited metformin and rosuvastatin plasma concentrations 1956 ± 93 ng mL^−1^ and 22.2 ± 0.4 ng mL^−1^, respectively.

## 3. Materials and Methods 

### 3.1. Chemicals and Reagents

Metformin hydrochloride of pharmaceutical purity grade was purchased from Wanbury Limited (BSEL Tech Park, Maharashtra, India). Rosuvastatin calcium of pharmaceutical purity grade was obtained from MSN Laboratories Private Limited (Rudraram, Telengana, India). The internal standard, N-despropyl ropinirole was purchased from Vitalife Chemipharma Pvt. Ltd. (Mumbai, Maharashtra, India). HPLC grade solvents were used and purchased from E. Merck (Darmstadt, Germany). A Synergy UV water purification system (Merck Millipore, Danvers, MA, USA) was used to provide water HPLC grade. Hydrophilic polytetrafluorethylene membrane syringe filters (PTFE, 13 mm, pore size 0.45 μm) were purchased from Merck Millipore (Danvers, MA, USA). Pooled drug-free human plasma was obtained from National Red Cross General Hospital, Athens, Greece.

### 3.2. Instrumentation

A single quadrupole mass spectrometer model Finnigan AQA (Thermo, Manchester, UK) equipped with an electrospray ionisation interface and an isocratic pump model Spectra Series P100 (ThermoSeparation, Piscataway, NJ, USA) LC system were used to perform the HILIC-ESI/MS experiments. Highly pure nitrogen was produced by using a Model Nitrox-N2, Domnick hunter (Gateshead, UK) nitrogen generator. Xcalibur software (v. 1.2, ThermoQuest, Austin, UK) was used for data acquisition and analysis. Metformin, rosuvastatin and N-despropyl ropinirol were separated using a Xbridge HILIC BEH analytical column (150.0 × 2.1 mm i.d., particle size 3.5 μm, 135 Å). To prolong column lifetime, a XBridge HILIC BEH guard cartridge (20 × 2.1 mm, 3.5 μm) was also used. A mobile phase comprising of 12% 15 mM ammonium formate water solution in acetonitrile and pumped at a flow rate of 0.25 mL min^−1^ was used to run the experiments. The mobile phase was always filtered prior to use through a 0.45 μm nylon-membrane filter, GelmanSciences (Northampton, UK) and degassed under vacuum. Chromatography was performed at 25 ± 2 °C with a chromatographic run time of 15 min. All analytes and the ISTD have been detected in electrospray positive ionization mode and quantitation was achieved in SIM mode. Source parameters were adjusted to the following settings: temperature 260 °C, capillary voltage 4.8 kV, cone voltage 20 V. 

### 3.3. Stock and Working Standard Solutions

Stock standard solutions of metformin and rosuvastatin were prepared at 500 μg mL^−1^ in acetonitrile-water mixture (90:10, *v*/*v*). These stock standard solutions were diluted in acetonitrile to prepare the mixed working standard solutions over the concentration ranges of 62.5 to 5000 ng mL^−1^ and 2 to 100 ng mL^−1^ for metformin and rosuvastatin, respectively. Stock standard solution of N-despropyl ropinirole (ISTD) was prepared at 190 μg mL^−1^ in acetonitrile. A working standard solution of ISTD at 3.8 μg mL^−1^ was prepared after further dilution in acetonitrile. The stock standard solutions were found to be stable for several months when stored at −20 °C. The working standard solutions were stored at 4 °C and prepared every month.

### 3.4. Calibration Standards and Quality Control Samples

Calibration standards spiked in human plasma were prepared at seven different concentration levels 62.5, 100, 250, 500, 1000, 2500 and 5000 ng mL^−1^ for metformin and at six different concentration levels 2, 5, 10, 20, 50 and 100 ng mL^−1^ for rosuvastatin. Each calibration sample contained 190 ng mL^−1^ of the internal standard. Quality control (QC) samples were prepared independently, in an analogous manner as the calibration standards, using separate stock solutions of the analytes. QC samples were prepared at three different concentration levels (62.5, 500 and 5000 ng mL^−1^) for metformin and (2, 10 and 100 ng mL^−1^) for rosuvastatin in human plasma.

### 3.5. Sample Preparation Procedure

Cleanup of biological samples is carried out by protein precipitation. On the day of extraction the samples are thawed at room temperature followed by vortex mixing to ensure homogeneity. Consequently, a 100 μL aliquot of each human plasma sample is transferred to a 2 mL Eppendorf tube, followed by addition of 50 μL of ISTD solution (3.8 μg mL^−1^), 30 μL of 5 mM ammonium formate solution in water and 820 μL of acetonitrile. The mixture is vortexed for 1.0 min and centrifuged at 16,000× *g* for 20 min at 25 °C. A 13 mm hydrophilic PTFE membrane syringe filter (pore size 0.45 μm) is then used to filter the samples prior to HILIC-ESI/MS analysis.

## 4. Conclusions

In this work the advantages of HILIC, on the separation of compounds with diverse physicochemical properties and on the improvement in ESI/MS sensitivity, were demonstrated through the development of a HILIC-ESI/MS for the quantitation of metformin and rosuvastatin in human plasma. For the first time metformin and rosuvastatin have been adequately retained and separated from matrix interferences with an analytical run time of 15 min by using a hydrophilic Xbridge^®^-HILIC BEH analytical column. The method is combined with a fast and simple procedure for sample preparation based on protein precipitation and filtration that requires only 100 μL of biological sample. Validation results demonstrate that under the optimum conditions metformin and rosuvastatin can be quantified accurately and precisely in human plasma. The method was successfully applied to the analysis of real samples and it can be used to support various clinical studies.

## Figures and Tables

**Table 1:** Analytical concentration parameters of the calibration equations for the determination of metformin and rosuvastatin in human plasma by HILIC-ESI/MS.

Compound	Concentration Range, ng mL^−1^	Regression Equations ^a^	r ^b^	Standard Deviation		S_r_^c^
				Slope	Intercept	
*Mean of three calibration curves over a period of one month*						
Metformin	62.5–5000	R_Mtf_ = 0.00715 × C_Mtf_ + 0.066	≥0.996	2.2 × 10^−4^	4.3 × 10^−4^	≤0.10
Rosuvastatin	2–100	R_Rsv_ = 0.01775 × C_Rsv_ − 0.0129	≥0.998	5.2 × 10^−4^	7.2 × 10^−3^	≤0.062

^a^ Ratios of the peak areas signals of metformin, R_Mtf_ and rosuvastatin, R_Rsv_, to that to the internal standard vs. the corresponding concentration of metformin, C_Mtf_ and rosuvastatin, C_Rsv_; ^b^ Correlation coefficient; ^c^ Standard error of the estimate.

**Table 2:** Data on accuracy and precision obtained from the analysis of quality control samples containing rosuvastatin and metformin (n = 3 runs; five replicates per run).

Compound	Concentration (ng mL^−1^)		
**Metformin**			
**Added Concentration**	**62.5**	**500**	**5000**
Run 1 (mean ± s.d.)	63.9 ± 6.1	489 ± 29	5045 ± 352
Run 2 (mean ± s.d.)	64.3 ± 5.1	42.9 ± 8.3	4698 ± 143
Run 3 (mean ± s.d.)	60.1 ± 6.0	488 ± 39	4768 ± 232
Overall mean	62.7	497.9	4837
Intra-day CV (%) ^a^	9.1	7.6	5.3
Inter-day CV (%) ^a^	1.8	1.0	3.0
Overall accuracy Er% ^b^	0.4	−0.4	−3.3
**Rosuvastatin**			
**Added Concentration**	**2**	**10**	**100**
Run 1 (mean ± s.d.)	2.31 ± 0.21	9.41 ± 0.33	104.1 ± 6.6
Run 2 (mean ± s.d.)	2.12 ± 0.22	9.61 ± 0.89	96.5 ± 5.7
Run 3 (mean ± s.d.)	2.01 ± 0.21	10.81 ± 0.46	96.2 ± 9.1
Overall mean	2.1	9.9	98.9
Intra-day CV (%) ^a^	8.5	6.2	7.3
Inter-day CV (%) ^a^	4.4	7.1	3.0
Overall accuracy Er% ^b^	7.0	−1.0	−1.1

^a^ Intra- and inter-assay coefficient of variations as calculated by ANOVA. ^b^ Relative percentage error.

**Table 3:** Recovery and ion suppression data for the quantitation of metformin and rosuvastatin by HILIC-ESI/MS.

Compound	Concentration Levels (ng mL^−1^)	
**Metformin**	**1000**	**5000**
% Recovery (mean ± s.d.)_n = 3_	92.6 ± 2.5	93.9 ± 1.4
% Matrix Factor (mean ± s.d.)_n = 3_	59.9 ± 1.0	63.7 ± 1.3
**Rosuvastatin**	**20**	**100**
% Recovery (mean ± s.d.)_n = 3_	96.6 ± 2.1	95.8 ± 4.1
% Matrix Factor (mean ± s.d.)_n = 3_	84.5 ± 7.1	81.3 ± 2.0
**N-despropyl ropinirole (ISTD)**	**3800**	
% Recovery (mean ± s.d.)_n = 3_	91.7 ± 3.8	
% Matrix Factor (mean ± s.d.)_n = 3_	61.8 ± 1.8	

**Table 4:** Stability data for metformin and rosuvastatin in human plasma under various storage conditions.

Compound	Calculated Concentration (ng mL^−1^)					
**Metformin**	**50**		**1250**		**2500**	
	**Mean ± S.D._(n = 3)_**	**%E_r_^a^**	**Mean ± S.D._(n = 3)_**	**%E_r_^a^**	**Mean ± S.D._(n = 3)_**	**%E_r_^a^**
Ambient temperature/4 h	50.4 ± 1.9	0.9	1271.5 ± 8.2	1.7	2522 ± 138	0.01
−20 °C/4 weeks	49.7 ± 2.1	−0.6	1260.1 ± 0.80	0.8	2412 ± 18	−0.04
−20 °C/4 Freeze-thaw cycles	51.21 ± 0.81	2.4	1282.3 ± 2.6	2.6	2386 ± 24	−0.05
**Rosuvastatin**	**2.5**		**25**		**50**	
Ambient temperature/4 h	2.509 ± 0.0089	0.4	24.72 ± 0.11	−1.1	49.4 ± 3.4	−0.9
−20 °C/4 weeks	2.457 ± 0.045	−1.7	24.53 ± 0.15	−1.9	50.2 ± 3.2	0.4
−20 °C/4 Freeze-thaw cycles	2.435 ± 0.031	−2.6	24.94 ± 0.42	−0.2	49.5 ± 2.4	−1.0

^a^ %E_r_: relative percentage error = (overall mean assayed concentration − added concentration)/(added concentration) × 100.

**Table 5:** Clinical data in patients treated with rosuvastatin and metformin.

Patient Sex/Age (years)	Time Post Dose (h)	Drug/Dose Peros (mg)	C (ng mL^−1^) Mean ± S.D._(n = 3)_	Co-Administered Drugs
♀/57	2_1/2_	Metformin/1000 × 1 × 30 days Rosuvastatin/10 × 1 × 30 days	1956 ± 93 22.2 ± 0.4	Acetylsalicylic acid 100 mg tb 1 × 1
♂/64	11	Rosuvastatin/20 × 1 × 30 days	16.2 ± 1.4	Furosemide 40 mg tb 1 × 1, ramipril 2.5 mg tb 1 × 1, carvedilol 6.35 mg tb 1 × 1.
♂/63	16	Rosuvastatin/20 × 1 × 30 days	9.21 ± 0.39	Nadroparin 5700 anti-ha 1 × 1 subcutaneous, omeprazole caps 20 mg 1 × 1, metoprolol tb 25 mg 1 × 2, sulbactam 1 g & ampicillin i.v. 1 × 3 × 5 days, acetyl salicylic acid 100 mg E.C.tb 1 × 1, atalopram 20 mg 1 × 1.
♀/80	2_1/2_	Metformin/1000 × 1 × 30 days	2635 ± 67	Acetylsalicylic acid 100 mg tb 1 × 1, insuline glarine 100 iu/mL 10IU ×1 night subcutaneous, Ramipril 5 mg tb 1 × 1, nadropanin 2850 Anti-xa 1 × 1, piracetam i.v. 1 × 2.
♀/83	10	Metformin/1000 × 2 × 30 days	1850 ± 55	Furosemide 20 mg tb 1 × 1, 1 × 1, insuline glarine 100 iu/mL 14IU ×1 night subcutaneous, pantoprazole 40 mg caps 1 × 1, T4-50 1 × 1, carvedilol 12.5 mg tb 1 × 2, apixaban 2.5 mg tb 1 × 2, Lutein/Zeaxanthine/Mesozeaxanthine caps 1 × 1, VITAMIN D3 2000 IU 1 × 1.
♂/70	2_1/2_	Metformin/850 × 2 × 30 days	1856 ± 84	Ezetimibe/simvastatin 10/40 mg tb 1 × 1, gliclazide 30 mg tb 1 × 1, sertraline 50 mg tb 1 × 2, galantamine 8 mg caps 1 × 1, levodopa/carvidopa/entacapone 100/25/200 tb 1 × 3, clopidogrel 75 mg tb 1 × 1.
♀/88	12	Metformin/850 × 1 × 30 days	1676 ± 60	Atorvastatin tb 40 mg 1 × 1, furosemide 20 mg i.v. 2 × 3, bisoprostol 10 mg 1 × 1, insuline glarine 100 iu/mL 10IU ×1 night subcutaneous, thyrormone 100 mg 1 × 1, acetyl salicylic acid 100 mg E.C.tb 1 × 1, digoxin tb 0.25 mg ½ × 1.
♀/88	16	Metformin/850 × 1 × 30 days	226 ± 12	Atorvastatin tb 40 mg 1 × 1, furosemide 20 mg i.v. 2 × 3, insuline glarine 100 iu/mL 10IU ×1 night subcutaneous, thyrormone 100 mg 1 × 1, digoxin tb 0.25 mg ½ × 1, acetyl salicylic acid 100 mg E.C. tb 1 × 1.
